# SAPAP3 regulates epileptic seizures involving GluN2A in post-synaptic densities

**DOI:** 10.1038/s41419-022-04876-9

**Published:** 2022-05-05

**Authors:** Yanke Zhang, Junhong Wu, Yin Yan, Yixue Gu, Yuanlin Ma, Min Wang, Hui Zhang, Kaiyan Tao, Yang Lü, Weihua Yu, Wei Jing, Xuefeng Wang, Xin Tian

**Affiliations:** 1 Department of Neurology, Chongqing Key Laboratory of Neurology, The First Affiliated Hospital of Chongqing Medical University, 1 Youyi Road, Chongqing, 400016 China; 2 Department of Neurology, Affiliated Hospital of Jining Medical University, Jining, Shandong China; 3 Department of Geriatrics, The First Affiliated Hospital of Chongqing Medical University, 1 Youyi Road, Chongqing, 400016 China; 4 Institute of Neuroscience, Chongqing Medical University, Chongqing, 400016 China

**Keywords:** Diagnostic markers, Epilepsy

## Abstract

Aberrantly synchronized neuronal discharges in the brain lead to epilepsy, a devastating neurological disease whose pathogenesis and mechanism are unclear. SAPAP3, a cytoskeletal protein expressed at high levels in the postsynaptic density (PSD) of excitatory synapses, has been well studied in the striatum, but the role of SAPAP3 in epilepsy remains elusive. In this study, we sought to investigate the molecular, cellular, electrophysiological and behavioral consequences of SAPAP3 perturbations in the mouse hippocampus. We identified a significant increase in the SAPAP3 levels in patients with temporal lobe epilepsy (TLE) and in mouse models of epilepsy. In addition, behavioral studies showed that the downregulation of SAPAP3 by shRNA decreased the seizure severity and that the overexpression of SAPAP3 by recombinant SAPAP3 yielded the opposite effect. Moreover, SAPAP3 affected action potentials (APs), miniature excitatory postsynaptic currents (mEPSCs) and N-methyl-D-aspartate receptor (NMDAR)-mediated currents in the CA1 region, which indicated that SAPAP3 plays an important role in excitatory synaptic transmission. Additionally, the levels of the GluN2A protein, which is involved in synaptic function, were perturbed in the hippocampal PSD, and this perturbation was accompanied by ultrastructural morphological changes. These results revealed a previously unknown function of SAPAP3 in epileptogenesis and showed that SAPAP3 may represent a novel target for the treatment of epilepsy.

## Introduction

Epilepsy, which is characterized by abnormal neuronal synchronization and spontaneous recurrent seizures (SRSs), is a devastating neurological disease with a prevalence of ~1–2% worldwide, and approximately one-third of individuals with epilepsy are resistant to common antiepileptic drugs (AEDs) [1]. Temporal lobe epilepsy (TLE) is the most common type of epilepsy, is characterized pathologically by the loss of hippocampal neurons and mossy fiber sprouting and is usually refractory to AEDs [2, 3]. Although the actual mechanism underlying epilepsy is unclear, an imbalance in synaptic excitatory and inhibitory transmission in the brain is one possibility. In particular, aberrant excitatory synaptic transmission may result in neuronal hyperexcitability and recurrent seizures [4–6]. Among neurons, synapses are neuronal contact sites that mediate the rapid transfer of information. The postsynaptic density (PSD), an electron-dense structure [7], is the most prominent postsynaptic component of excitatory synapses [8] and is composed of many macromolecular complexes and scaffolding proteins. Therefore, the PSD is the focus of investigations of synaptic function.

Synapse-associated protein 90/PSD-95-associated proteins (SAPAPs), including SAPAP1, SAPAP2, SAPAP3, and SAPAP4, are adapter proteins located in the PSD [9]. These synaptic scaffolding proteins associate with different synaptic scaffolding/cytoskeletal proteins and signaling components, which are thus believed to assemble functional multiprotein units at synapses [10, 11]. Of the four family members, SAPAP3 is the only protein that is highly expressed in the striatum and participates in direct interactions with PSD95 family proteins, which are thought to function in multiprotein units as linker proteins between the glutamate receptor and the cytoskeleton [12]. In addition, mice with a genetic deletion of SAPAP3 exhibit increased behaviors, including anxiety and compulsive grooming [12]. More interestingly, recent evidence from human genetic studies also supports a role for SAPAPs in obsessive-compulsive behaviors [13, 14]. Shank proteins are synaptic cytoskeleton-associated proteins that interact with SAPAPs, and previous studies identified a role for Shank3 in epilepsy [15–17] and indicate that PSD95/SAPAP/Shank multiprotein units might play an important role in organizing the postsynaptic signaling complex at glutamatergic synapses [18, 19]. Few studies have investigated SAPAPs in the hippocampus; however, a previous study showed that SAPAP1, SAPAP2, and SAPAP4 transcripts are distributed in the cell body and that SAPAP3 is found in the molecular layers, which indicates that SAPAP3 may contribute to the function of dendritic spines in the hippocampus [20]. Thus, SAPAP3 might contribute to the establishment of the neuronal architecture in the hippocampus, but this hypothesis requires further investigation.

Previous reviews reported that patients with TLE exhibit higher lifetime frequencies of psychiatric disorders (70%) and obsessive-compulsive disorder (OCD; 11.0%), but the intersections between TLE and psychiatric disorders remain obscure [21]. Based on SAPAP3genetic studies of SAPAP3 and the crucial role of SAPAP3 in excitatory receptor function, we hypothesized that SAPAP3 may participate in the pathology and development of epilepsy. Here, SAPAP3 expression was detected in patients with epilepsy and animal models, and the detailed relationship between the excitability of brain slices and NMDARs on PSD in relation to SAPAP3 was investigated. The present study mainly aimed to elucidate the possible relationship between SAPAP3 and epilepsy.

## Materials and methods

### Subjects

Adult male mice (aged 7–8 weeks; 22–24 g) were used in the current experiments. The animals were housed (5 per cage) in a climate-controlled environment with a 12-h light/12-h dark cycle. The mice had free access to food and water. All experiments were conducted during the light phase.

Twenty experimental samples and ten control samples were randomly selected from our human brain bank. A neurological examination, detailed history, electroencephalogram (EEG) studies and neuroradiological studies were performed prior to surgery. All patients were diagnosed by two or more neurologists according to the standards established by the International League Against Epilepsy (1981). All patients were diagnosed with refractory epilepsy and required surgery. The clinical manifestations of the patients with TLE and control subjects are summarized in Supplementary Table 1.

### Lentiviral vector construction, microinjections and validation

For shRNA-mediated knockdown and recombinant SAPAP3 overexpression, lentivirus (LV) construction and production were performed by Shanghai Genechem Co., Ltd. (Shanghai, China). An shRNA oligonucleotide was cloned into the U6-shRNA-Ubi-EGFP vector to construct the LV for the knockdown of SAPAP3 expression. Different shRNAs targeting mouse SAPAP3 genes and recombinant SAPAP3 were screened and confirmed in vivo. The second SAPAP3-sh (shown in Supplementary Table 2), which effectively knocked down SAPAP3 expression, was selected. For recombinant SAPAP3 overexpression, information about the SAPAP3 gene was obtained from NM_198618, and the carrier was pGC-FU-Ubiquitin Promoter-3FLAG-SV40 Promoter-EGFP-IRES-puromycin. More information is shown in Supplementary Table 2.

The mice were randomly divided into four groups: SAPAP3-sh-con group, SAPAP3-sh group, SAPAP3-overexpression group and SAPAP3-con group. We administered bilateral microinjections of a total volume of 4 μl (2 μl per side, 1 μl per site) of LV into the hippocampus of each mouse using a glass pipette (0.2 μl/min; location: anteroposterior (AP) = 2 mm, mediolateral (ML) = 1.5 mm, dorsoventral (DV) = 1.5 mm; and AP = 2 mm, ML = 2 mm, DV = 2 mm). The pipette was left in place for at least 5 min after the injection to prevent backflow. To confirm the infection efficiency, samples were obtained 3 or 7 days after virus administration for western blotting and immunofluorescence.

### Mouse model construction and behavioral assay

The pilocarpine-induced epileptic mouse model was established as previously reported [22]. To augment the effects of the infection, we conducted behavioral tests 10 days after administration of the lentiviral vectors. In brief, the mice which were successfully infected with the LV were administered an intraperitoneal (i.p.) injection of 1 mg/kg methyl-scopolamine followed by an i.p. injection of 320 mg/kg pilocarpine (Sigma, St. Louis, MO, USA) 30 min later to induce the onset of status epilepticus (SE). The mice were monitored continuously by a video recording system 24 h/day for 30 consecutive days after termination of the pilocarpine-induced SE with diazepam (10 mg/kg, i.p.; Sigma–Aldrich Co., St. Louis, MO, USA). In the chronic period, the severity of seizures was scored using the Racine scale [23] and assessed by two double-blind investigators. The mice were sacrificed on the 35th day after SE to study bellowing. The latency time was defined as the time from the pilocarpine injection to the onset of the first SRSs (rated 4 or 5). The induction of SE by pilocarpine and the epilepsy model were confirmed by EEGs.

For the pentylenetetrazole (PTZ) chronic kindling model, the mice were administered a subconvulsive dose of PTZ (35 mg/kg, i.p.; Sigma–Aldrich Co., St. Louis, MO, USA) from 8:00 to 12:00 a.m. every day for 30 consecutive days using methods described by Maciejak et al. [24]. After each PTZ injection, the convulsive behavior was observed for at least 30 min and recorded according to the Racine scale [23]. The animals were considered fully kindled after experiencing three consecutive class 4 or 5 seizures. The latency time was defined as the last day of three consecutive class 4 and 5 seizures.

Epileptic mouse brain tissues were obtained from pilocarpine-induced epileptic mice with SRSs or mice fully kindled after PTZ treatment. Correspondingly, the control samples used in this study originated from mice injected with saline.

### Electrophysiology

In vivo electrophysiological recordings were obtained using a previously described protocol [25]. A microwire array (a 2 × 8 array of platinum-iridium alloy wire, each with a diameter of 25 μm) was implanted into the right hippocampus (location: AP = 2 mm, ML = 1.5 mm, DV = 1.5 mm) to record the hippocampal EEG. All accessories were attached to the skull with dental cement. The animals were allowed to recover from surgery for 7 days and to adapt to the environment for 3 days prior to the recordings. EEGs were filtered (0.1–1000 Hz), amplified 1000×, and digitized at 4 kHz using an OmniPlex® D Neural Data Acquisition System (Plexon, Dallas, TX) as previously described. All recordings were referenced to two screws implanted on the skull. NeuroExplorer® v4.0 (Plexon, Dallas, TX) was used for the analysis.

For the in vitro electrophysiological recordings, 7- to 8-week-old mice were successfully infected with SAPAP3-sh, SAPAP3-sh-con, recombinant SAPAP3 or SAPAP3-con. Coronal slices (thickness of 300 μm) were cut using a vibratome (Leica VT1200S). The slices were cut in ice-cold solutions containing 60 mM NaCl, 100 mM sucrose, 2.5 mM KCl, 1.25 mM NaH_2_PO_4_•2H_2_O, 20 mM D-glucose, 26 mM NaHCO_3_, 1 mM CaCl_2_, and 5 mM MgCl_2_•6H_2_O saturated with 95% O_2_ and 5% CO_2_. The slices were allowed to recover in artificial cerebral spinal fluid (ACSF, pH 7.4) for 1 h at 32 °C with 125 mM NaCl, 3 mM KCl, 1.25 mM NaH_2_PO_4_•2H_2_O, 15 mM D-glucose, 26 mM NaHCO_3_, and 2 mM CaCl_2_ saturated with 95% O_2_/5% CO_2_. In the whole-cell recording experiments, pyramidal neurons in the hippocampal CA1 area were selected for recording under an inverted phase contrast microscope (Nikon, Japan), and the slices were fully submerged in Mg^2+^-free ACSF (4 ml/min) at room temperature (20 to 25 °C). The access resistance was maintained at less than 20 MΩ. The series resistance (15–30 MΩ) was compensated automatically using a MultiClamp 700B and was monitored throughout the recording. Cells were discarded if the series resistance changed by more than 20% of the initial value. Data were collected after the current was stable for 5 to 15 min.

Epileptic discharges were characterized by action potentials (APs) manifested as continuous high-frequency spike discharges. Glass pipettes were filled with the following internal solution to measure the APs: 60 mM K_2_SO_4_, 60 mM N-methyl-D-glucamine, 40 mM HEPES, 4 mM MgCl_2_•6H_2_O, 0.5 mM BAPTA, 12 mM phosphocreatine, 2 mM Na_2_ATP, and 0.2 mM Na_3_GTP (pH 7.4 adjusted with KOH). Glass microelectrodes were filled with the following solution to measure the miniature excitatory postsynaptic currents (mEPSCs): 130 mM CsMeSO_4_, 10 mM CsCl_2_, 10 mM HEPES, 4 mM NaCl, 1 mM MgCl_2_, 1 mM EGTA, 5 mM MgATP, 0.5 mM Na_3_GTP, 12 mM phosphocreatine, and 5 mM N-methyl-d-glucamine (NMG) (pH adjusted to 7.2 with CsOH, 275–290 mOsm). Picrotoxin (PTX, 100 μM) and tetrodotoxin (TTX, 1 μM) were added to Mg^2+^-free ACSF to record the mEPSCs. For the miniature inhibitory postsynaptic current (mIPSC) recordings, the internal solutions in the glass microelectrodes contained 100 mM CsCl, 10 mM HEPES, 1 mM MgCl_2_, 1 mM EGTA, 5 mM MgATP, 0.5 mM Na_3_GTP, 12 mM phosphocreatine, and 30 mM NMG (pH adjusted to 7.2 with CsOH, 275–290 mOsm). The slices were submerged and continuously perfused with Mg^2+^-free ACSF containing 6,7-dinitroquinoxaline-2,3(1H,4H)-dione (DNQX, 20 μM), DL-2-amino-5-phosphonovalerate (D-APV, 50 μM) and TTX (1 μM). The membrane potentials were maintained at −70 mV in the voltage-clamp mode.

The evoked EPSCs were recorded in the presence of 100 μM PTX, glass microelectrodes were filled with the same solution used to record the mEPSCs. α-Amino-3-hydroxy-5-methyl-4-isoxazole propionic acid receptor (AMPAR)- and N-methyl-D-aspartate receptor (NMDAR)-mediated synaptic responses were evoked by a bipolar stimulation electrode located in the Schaffer collaterals ~50 μm rostral to the recording electrode in the same layer. First, we obtained stable synaptic responses at −70 mV; the amplitude of these responses was recorded as the AMPAR-mediated EPSCs. Second, the holding potential was changed to +40 mV, the amplitude of the NMDAR-mediated EPSCs obtained at 50 ms after the stimulus. For the paired-pulse ratio (PPR), the holding potential was −70 mV in the presence of 100 μM PTX. The interval for paired stimulations was set to 50 ms. The PPR was defined as the second peak amplitude (P2) divided by the first peak amplitude (P1).

Signals were acquired using a MultiClamp 700B amplifier (Axon, USA) and recorded with pClamp 10.3 software (Molecular Devices). All recordings were filtered at 2 kHz, digitized at 10 kHz (DigiData 1440A and pClamp 10.3, Molecular Devices), and then analyzed using Mini Analysis Software (Synaptosoft, Leonia, NJ, USA) and Clampfit 10.3 software (Molecular Devices).

### Sample preparation

The mice were deeply anesthetized with 1% sodium pentobarbital prior to decapitation and tissue extraction. Half of mice or human brain tissues was placed in liquid nitrogen for western blotting, and the other half was embedded in 4% paraformaldehyde for immunofluorescence. Transmission electron microscopy was conducted according to standard protocols.

### Preparation of subcellular fractions and western blots

Subcellular fractions were obtained as previously reported [26, 27]. Equivalent amounts (total proteins, 50 μg; subcellular fractions, 10 μg) of the samples were separated on 10–12% SDS-PAGE gels and then transferred onto PVDF membranes. The membranes were incubated with 5% nonfat milk for 1 h at room temperature. The target proteins were blotted with primary antibodies against SAPAP3 (1:200, Proteintech), GluN1 (1:1000, Abcam), GluN2A (1:1000, Millipore), GluN2B (1:1000, Millipore), and GAPDH (1:5000, Abcam) overnight at 4 °C. On the following day, the blots were incubated with HRP-conjugated secondary antibodies (1:3000, room temperature for 1 h). An enhanced chemiluminescence detection system (Amersham ECL) and the Bio-Rad ChemiDoc XRS + system were used to detect and quantify the bands. Densitometry was performed using Quantity One Software (Bio-Rad Laboratories, Hercules, USA). The total protein samples were normalized to GAPDH expression, and the proteins in the subcellular fractions were normalized to the total protein samples.

### Immunofluorescence

Immunofluorescence was performed as previously described [27]. Tissue sections (10 μm) were incubated in normal goat serum (Zhongshan Golden Bridge, Beijing, China) for 60 min and then incubated with a mixture containing SAPAP3 (rabbit polyclonal antibody, 1:100, Proteintech), microtubule-associated protein 2 (MAP2) (chicken polyclonal antibody, 1:100, Abcam), and glial fibrillary acidic protein (GFAP) antibodies (mouse polyclonal antibody, 1:100, Zhongshan Golden Bridge) or a mixture containing SAPAP3, PSD95 (mouse polyclonal antibody, 1:100, Millipore) and VGLUT1 antibodies (guinea pig polyclonal antibody, 1:100, Synaptic Systems) overnight at room temperature. After washing with phosphate-buffered saline (PBS), the sections were incubated with a mixture containing DyLight 488-conjugated AffiniPure goat anti-rabbit IgG (1:200, Zhongshan Golden Bridge), DyLight 405-conjugated AffiniPure goat anti-chicken IgG (1:200, Abcam), DyLight 647-conjugated AffiniPure goat anti-guinea pig IgG (1:200, ImmunoReagents) and DyLight 594-conjugated AffiniPure goat anti-mouse IgG (1:200, Zhongshan Golden Bridge) in a dark room for 60 min at 37 °C and then mounted in 50% glycerol/PBS. Fluorescence was detected using a laser-scanning confocal microscope (Leica Microsystems Heidelberg GmbH, Germany) with an Olympus IX 70 inverted microscope (Olympus, Japan) equipped with a FluoView FVX confocal scan head.

### Transmission electron microscopy

Transmission electron microscopy was performed as previously described [28, 29] with some modifications. After behavioral testing, the mice were anesthetized and transcardially perfused with 0.9% NaCl and then with 2% paraformaldehyde plus 2.5% glutaraldehyde in 0.1 M PBS (pH 7.2). The hippocampal CA1 region was dissected from the appropriate sections and then trimmed to obtain ~1-mm^3^ trapezoids. The slabs were submerged in 4% glutaraldehyde (0.1 M sodium cacodylate buffer, pH 7.2), trimmed and embedded in Spurr’s medium. The tissues were postfixed with osmium. Ultrathin sections (80 nm) were cut with an ultramicrotome, collected on copper grids, stained with 4% uranyl acetate and lead citrate and observed on 300-mesh coated grids using a JEOL JEM-2100F (Tokyo, Japan) transmission electron microscope. Asymmetric (excitatory) nonperforated synapses on dendritic spines in the CA1 region with well-defined presynaptic compartments, clear synaptic clefts and postsynaptic densities were selected and photographed at 100 kV and a final magnification of 25,000 or 50,000. The electron micrographs were analyzed by 2 observers who were blinded to the treatment groups. The synapse density refers to the number of synapses per 30 μm^2^. The thickness of the PSD was also measured.

### Statistical analyses

The data are presented as the means ± SEM. The samples were analyzed in independent triplicates. The data from more than two groups (SPSS 19.0) were compared by one-way ANOVA followed by the post hoc test. Independent samples Student’s *t* tests were used to compare two groups. The *χ*^2^ test was used to compare gender differences. Group differences in the mean seizure score during PTZ-kindling were evaluated with repeated measures ANOVA. Nonparametric tests were conducted when appropriate. *p* < 0.05 indicated statistical significance.

## Results

### SAPAP3 expression is significantly increased in the cortex of patients with TLE

**Fig. 1:**
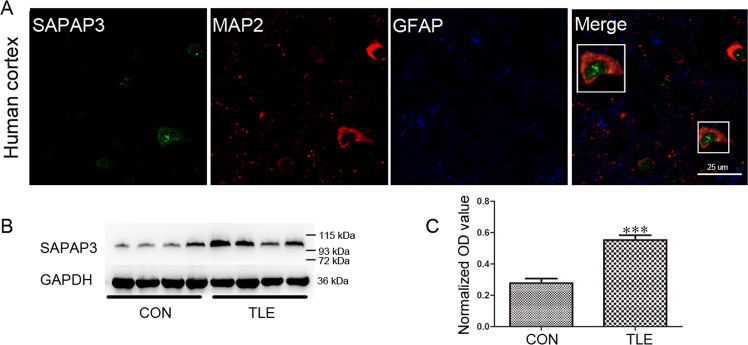
Expression of SAPAP3 in the temporal cortex of patients with TLE. **A** Triple-label immunofluorescence showed that SAPAP3 (green) and GFAP (blue) were not coexpressed in astrocytes, but SAPAP3 (green) and MAP2 (red) were coexpressed in the cortical neurons of patients. The arrows indicate positive cells. **B** Representative western blots showing the expression of SAPAP3 in patients with TLE and controls. **C** The comparison of the mean OD value indicates that SAPAP3 expression in the TLE group (*n* = 20) is significantly higher than that in the control group (*n* = 10). Data are presented as means ± SEM. ***p < 0.001.

The TLE group included 20 patients (11 males and 9 females) with a mean age of 30.30 ± 1.70 years and an average disease course of 12.40 ± 1.10 years. The control group included 10 subjects (5 females and 5 males) with a mean age of 26.60 ± 2.90 years. No significant differences in age or sex were found between the TLE and control groups (*p* > 0.05). The SAPAP3 expression patterns in patients with TLE were investigated by immunofluorescence and western blotting. In the temporal cortex of patients with TLE, SAPAP3 (green) was coexpressed with MAP2 (red) within neurons but not with GFAP (blue) in astrocytes (Fig. 1A). The expression levels of SAPAP3 in patients with TLE were confirmed by western blotting, and the results showed that the normalized optical density (OD) of SAPAP3 was 0.27 ± 0.02 in the control group and increased significantly to 0.55 ± 0.03 in the TLE group (*p* < 0.001; Fig. 1B, C). SAPAP3 expression was normalized by calculating the ratio of the OD of SAPAP3 to that of GAPDH.

### SAPAP3 is expressed in excitatory synapses in the mouse epilepsy model

**Fig. 2:**
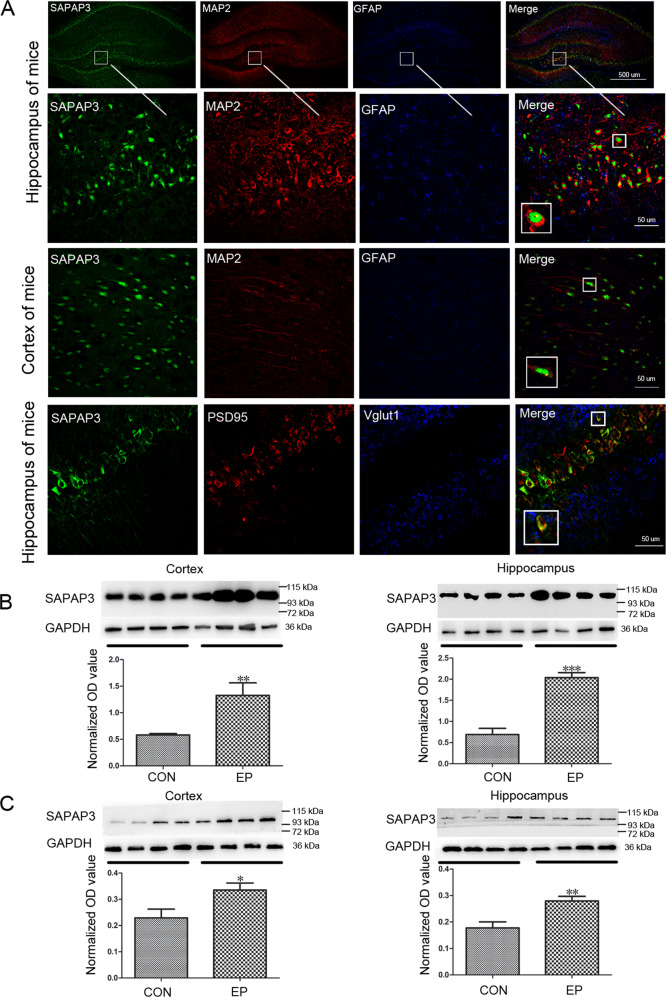
SAPAP3 expression in the hippocampus and cortex of the mouse model. **A** Triple-label immunofluorescence showed that SAPAP3 (green) and GFAP (blue) were not coexpressed in astrocytes, but SAPAP3 (green) and MAP2 (red) were mainly coexpressed within mouse hippocampal (upper panel) and cortical neurons (middle panel). Triple-label immunofluorescence showed that SAPAP3 (green) and VGLUT1 (blue) were not coexpressed, but SAPAP3 (green) and PSD95 (red) were coexpressed (lower panel). **B** Representative western blot images and quantification of SAPAP3 expression showed a significant increase in SAPAP3 expression in the pilocarpine-induced mouse epilepsy model compared with controls (left panel, cortex; right panel, hippocampus). **C** Representative western blot images and quantification of SAPAP3 expression showed a significant increase in SAPAP3 expression in the PTZ-induced mouse epilepsy model compared with the controls (left panel, cortex; right panel, hippocampus). Data are presented as means ± SEM, *n* = 12 per group. *p < 0.05; **p < 0.01; ***p < 0.001.

Although SAPAP3 expression was upregulated in human patients, we obtained more details using an animal model. The hippocampus is considered the core source of epilepsy, and because hippocampal tissues cannot be obtained from patients and controls, we tested the expression patterns of SAPAP3 in the hippocampus and cortex of a mouse model of pilocarpine-induced epilepsy to confirm the expression of SAPAP3 in patients and to exclude the possible effect of AEDs. Consistent with the results from the patients, immunofluorescence staining results obtained with the mouse model showed that SAPAP3 (green) was mainly coexpressed with MAP2 (red) within hippocampal and cortical neurons but not with GFAP (blue) in astrocytes (Fig. 2A). As shown in Fig S1, SAPAP3 (green) was mainly coexpressed with MAP2 (red) within hippocampal and cortical neurons in the control mouse brain but not with GFAP (blue) in astrocytes. In addition, SAPAP3 was coexpressed with PSD95 (lower panel in Fig. 2A), which indicated that SAPAP3 was located in the PSD of excitatory synapses. As shown in the western blots, SAPAP3 was upregulated in the mouse model of pilocarpine-induced epilepsy (cortex: epilepsy group, 1.32 ± 0.23 vs. the control group, 0.58 ± 0.03; *p* < 0.01; hippocampus: epilepsy group, 2.04 ± 0.12 vs. the control group, 0.69 ± 0.15; *p* < 0.001; Fig. 2B). The same results were observed in the hippocampus and cortex of the mouse PTZ-kindling model of epilepsy (cortex: epilepsy group, 0.34 ± 0.03 vs. the control group, 0.23 ± 0.03; *p* < 0.05; hippocampus: epilepsy group, 0.28 ± 0.02 vs. the control group, 0.18 ± 0.02; *p* < 0.01; Fig. 2C).

### Confirmation of the efficiency of lentivirus infection

Laser-scanning confocal fluorescence microscopy was performed to detect the area in which the infected GFP cDNA was expressed and to confirm the infection efficiency of the LV. On the 3rd day after LV injection, the green fluorescence intensity was weak (data not shown), and on the 7th day, GFP expression was obvious and showed widespread infection in the hippocampus (Fig. S2A). The levels of the SAPAP3 protein on the 7th day were detected by western blotting to confirm the inhibition and overexpression efficiencies. The levels of the SAPAP3 protein in the SAPAP3-sh group were significantly lower than those in the SAPAP3-sh-con group (0.19 ± 0.03 vs. 0.46 ± 0.05; *p* < 0.01; Fig. S2B, C). In contrast, the SAPAP3 protein levels in the SAPAP3-overexpression group were significantly higher than those in the SAPAP3-con group (1.41 ± 0.08 vs. 0.53 ± 0.04; *p* < 0.001; Fig. S2B, C).

### Effect of SAPAP3 on epileptic seizures

**Fig. 3:**
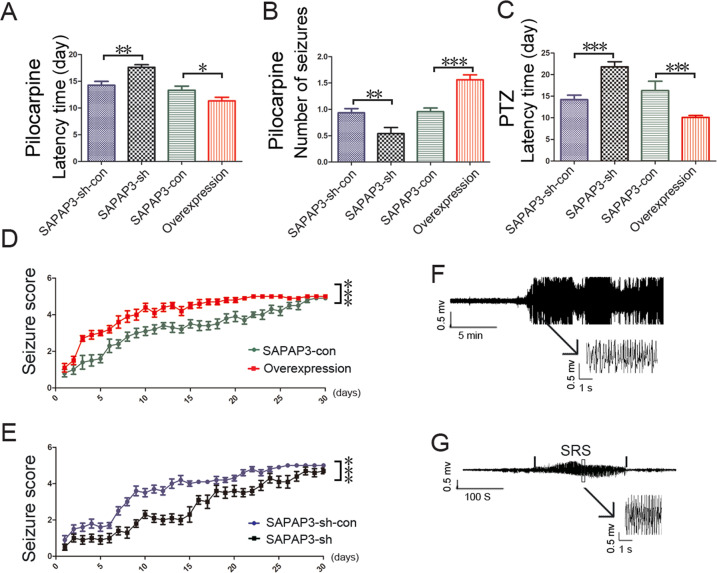
Seizure-modifying effects of SAPAP3 on the pilocarpine-induced model and PTZ-kindling model. **A** SAPAP3-sh treatment significantly increased the latency time to the first spontaneous seizures and **B** reduced the numbers of pilocarpine-induced spontaneous seizures in the chronic period. Recombinant SAPAP3 yielded the opposite results (**A**, **B)**. Data are presented as means ± SEM, *n* = 8–10. *p < 0.05; **p < 0.01; ***p < 0.001. **C** SAPAP3-sh treatment significantly increased the latency time in the pentylenetetrazol-induced kindling model and **E** reduced the severity of pentylenetetrazol-induced seizures. Recombinant SAPAP3 yielded the opposite results (**C**, **D**). Data are presented as means ± SEM, *n* = 10. ***p < 0.001. **F** Representative EEG recording of pilocarpine-induced status epilepticus. **G** Typical EEG showing that mice with spontaneous recurrent seizures exhibited epileptic discharges.

The mice in all groups injected with LV did not exhibit spontaneous seizures, epileptiform discharge or OCD phenotypes (data not shown). The expression of SAPAP3 is increased in epilepsy, but the effect of SAPAP3 on epilepsy is unknown. We constructed a mouse pilocarpine-induced epilepsy model to examine the role of SAPAP3 in the development of epilepsy. In the behavioral tests, the latency time of the SAPAP3-sh-con group was 14.25 ± 0.73 days, but a longer latency time of 17.60 ± 0.50 days was found for the SAPAP3-sh group (*p* < 0.01; Fig. 3A). In contrast, the latency time of the SAPAP3-overexpression group was 11.33 ± 0.67 days, and the SAPAP3-con group exhibited a longer latency time of 13.33 ± 0.76 days (*p* < 0.05; Fig. 3A). The frequency of SRSs is partially related to the disease severity of the mouse epilepsy model. Thus, we observed the mean number of SRSs experienced by each mouse. The mean number of SRSs per mouse in the SAPAP3-sh group per day was 0.54 ± 0.12, and a significant increase was observed in the SAPAP3-sh-con group (0.93 ± 0.08; *p* < 0.01; Fig. 3B). In contrast, the mean number/mouse/day obtained for the SAPAP3-con group was 0.96 ± 0.07, and a significant increase was found in the SAPAP3-overexpression group (1.56 ± 0.09/mouse/day; *p* < 0.001; Fig. 3B). EEG was used to confirm the induction of SE by pilocarpine (Fig. 3F) and that mice were experiencing SRSs (Fig. 3G).

Another animal model, the chronic PTZ-kindling mouse model, was selected to confirm the effect of SAPAP3 on epilepsy. The latency time of the SAPAP3-sh-con and SAPAP3-sh groups was 14.20 ± 1.04 and 21.80 ± 1.19 days, respectively (*p* < 0.001; Fig. 3C), and SAPAP3 overexpression led to the opposite results (SAPAP3 overexpression group: 10.10 ± 0.43 days vs. SAPAP3-con group: 16.30 ± 2.16 days; *p* < 0.001; Fig. 3C). The severity of general tonic clonic seizures was enhanced by recombinant SAPAP3 (*p* < 0.001; Fig. 3D) and attenuated by SAPAP3-sh (*p* < 0.001; Fig. 3E) compared with the degrees of severity observed in their respective control groups.

As demonstrated by both animal behavior tests, the latency time was longer in the SAPAP3-sh group and shorter in the SAPAP3-overexpression group compared with the respective control groups, which was consistent with our hypothesis. SAPAP3 in the mouse hippocampus might participate in the process of epilepsy.

### Effects of SAPAP3 on NMDAR-mediated excitatory synaptic transmission

**Fig. 4:**
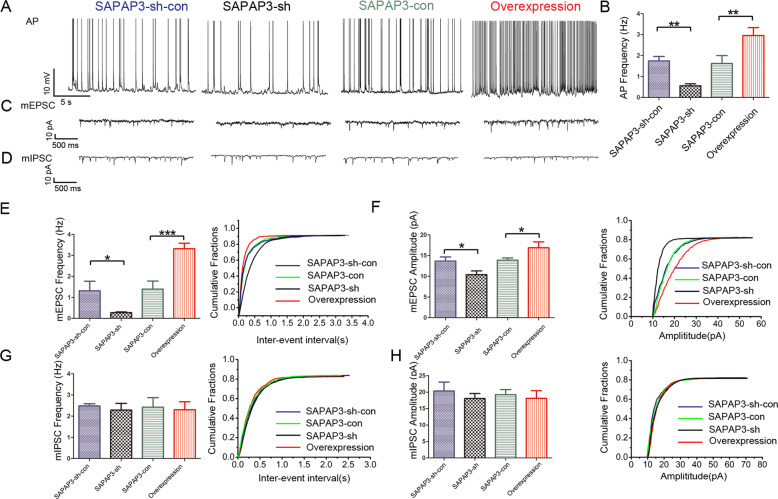
Electrophysiological changes in hippocampal neurons in slices after SAPAP3 knockdown and overexpression. **A**, **C**, **D** Representative traces of APs, mEPSCs and mIPSCs in slices from mice treated with SAPAP3-sh-con, SAPAP3-sh, SAPAP3-con and recombinant SAPAP3 recorded in Mg^2+^-free ACSF. **B** Compared with the SAPAP3-sh-con group, the SAPAP3-sh LV significantly decreased the average frequency of APs. Compared with the SAPAP3-con group, the SAPAP3 overexpression LV significantly increased the average frequency of APs. SAPAP3-sh LV treatment significantly decreased the average frequency (**E**) and amplitude (**F**) compared with those of the control group and its representative cumulative fractions. SAPAP3 overexpression LV treatment yielded the opposite results (**E**, **F**). The data did not show a significant difference in the frequency (**G**) and amplitudes (**H**) of the mIPSCs among the groups. Data are presented as means ± SEM, *n* = 6 per group. *p < 0.05; **p < 0.01; ***p < 0.001.

**Fig. 5:**
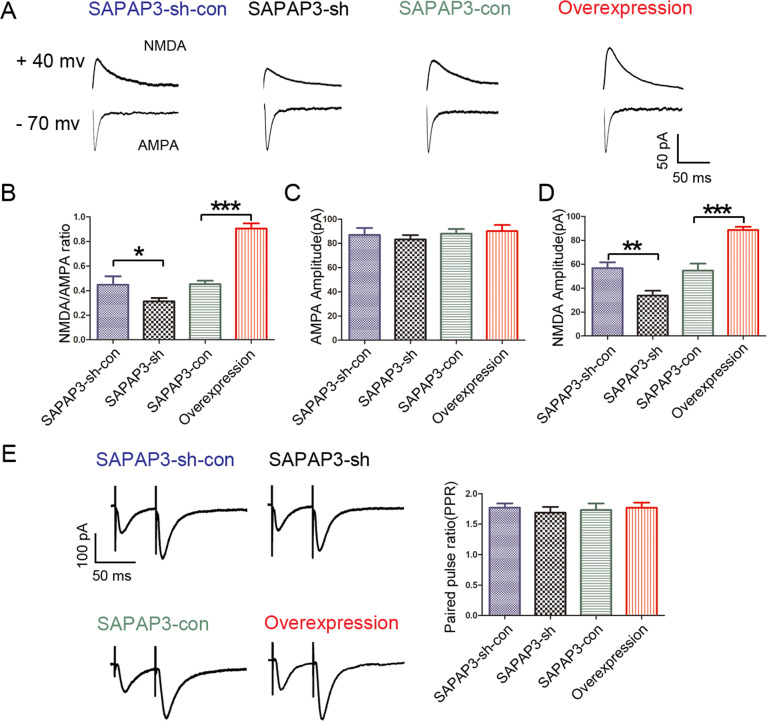
Perturbations in the evoked EPSCs in response to changes in the SAPAP3 levels in the different groups. **A** Representative traces of evoked AMPAR- (−70 mV) and NMDAR-mediated (+40 mV) EPSCs; NMDA receptor-mediated responses were measured at 50 ms poststimulus. **B** Summary graph showing that the NMDA/AMPA ratio was lower in the SAPAP3-sh-treated mice than in the controls. SAPAP3 overexpression from the recombinant SAPAP3 LV yielded the opposite results. **C** The SAPAP3-sh and SAPAP3 overexpression LV affected AMPAR-mediated EPSCs, but the difference was not significant. **D** The SAPAP3-sh LV significantly decreased the average amplitude of NMDAR-mediated EPSCs compared with that of the control group; SAPAP3 overexpression yielded the opposite results. **E** No significant difference in the PPRs for AMPAR-mediated EPSCs was found among the groups. Data are presented as means ± SEM, *n* = 6 per group. *p < 0.05; **p < 0.01; ***p < 0.001.

We applied a whole-cell patch-clamp electrophysiological approach to CA1 pyramidal neurons using hippocampal slices from mice that had been successfully infected with recombinant SAPAP3, SAPAP3-con, SAPAP3-sh or SAPAP3-sh-con LV to further evaluate the role of SAPAP3 in acute brain slices and verify the observations from animal behavioral tests. The perfusion of hippocampal slices with Mg^2+^-free ACSF induced bursts of APs, which are indicative of the successful induction of epileptiform activity. Slices of the SAPAP3-sh group displayed significantly lower frequencies of APs (0.56 ± 0.09 Hz) than the slices of the SAPAP3-sh-con group (1.75 ± 0.21 Hz; *p* < 0.01; Fig. 4A, B) under the same conditions. In contrast, SAPAP3 overexpression yielded the opposite effect (SAPAP3 overexpression group: 2.95 ± 0.38 Hz vs. SAPAP3-con group: 1.62 ± 0.37 Hz; *p* < 0.01; Fig. 4A, B). The hyperexcitability of neurons is often caused by an imbalance between excitatory and inhibitory transmission. In addition, according to our previous study, SAPAP3 is mainly located in excitatory synapses. We recorded mEPSCs and mIPSCs to further examine the excitatory/inhibitory balance. The frequency of mEPSCs was significantly lower in the SAPAP3-sh group than in the SAPAP3-sh-con group (0.28 ± 0.03 Hz vs. 1.31 ± 0.45 Hz; *p* < 0.05; Fig. 4C, E), and recombinant SAPAP3 had the opposite effect (SAPAP3-con group: 1.39 ± 0.38 Hz vs. SAPAP3-overexpression group: 3.31 ± 0.27; *p* < 0.001; Fig. 4C, E). The amplitudes of the mEPSCs were significantly affected by SAPAP3 (SAPAP3-sh-con group: 13.68 ± 0.98 pA vs. SAPAP3-sh group: 10.40 ± 0.89 pA; *p* < 0.05; SAPAP3-con group: 13.89 ± 0.56 pA vs. SAPAP3-overexpression group: 16.88 ± 1.41 pA; *p* < 0.05; Fig. 4C, F). No significant differences in the frequencies or amplitudes of the mIPSCs were found among the groups (*p* > 0.05; Fig. 4D, G, H). Thus, hyperexcitability might be partially attributed to abnormal excitatory synaptic transmission. The prevailing hypothesis states that AMPA-type glutamate receptors (AMPARs) and NMDA-type glutamate receptors (NMDARs) are fundamentally needed for information processing in the brain. We then recorded the evoked EPSCs to expound on this hypothesis and explored whether the altered neuronal excitability was caused by NMDAR-mediated or AMPAR-mediated currents. The results showed that the NMDA/AMPA ratio of the SAPAP3-sh group was significantly lower than that of the SAPAP3-sh-con group (0.31 ± 0.03 vs. 0.45 ± 0.07; *p* < 0.05; Fig. 5A, B). In addition, the amplitude of NMDAR-mediated currents was significantly decreased in the SAPAP3-sh group (SAPAP3-sh group: 33.91 ± 3.90 vs. the SAPAP3-sh-con group: 56.75 ± 4.89; *p* < 0.01; Fig. 5A, D), and some changes in the amplitudes of APMAR-mediated currents were detected; however, the differences in amplitude between the SAPAP3-sh and SAPAP3-sh-con groups were not significant (*p* > 0.05; Fig. 5A, C). Recombinant SAPAP3 yielded the opposite results (*p* < 0.001; Fig. 5A, B; *p* < 0.001; Fig. 5A, D). Neuronal excitability is mainly controlled by presynaptic excitatory neurotransmitter release and postsynaptic excitatory receptor function [30, 31]. To investigate whether the effect of SAPAP3 on synaptic responses occurs presynaptically or postsynaptically, the PPRs of EPSCs were recorded, and no significant differences were detected among the intervention groups (*p* > 0.05; Fig. 5E), which indicated that SAPAP3 may modulate glutamatergic transmission through a postsynaptic rather than a presynaptic mechanism.

Thus far, our findings of an altered NMDA/AMPA ratio, regulation of the mEPSC amplitude/frequency and normal presynaptic release likely indicate that the perturbations in postsynaptic efficacy are attributable to an imbalance in the SAPAP3 levels.

### Behavioral performance accompanies GluN2A perturbations in the PSD

**Fig. 6:**
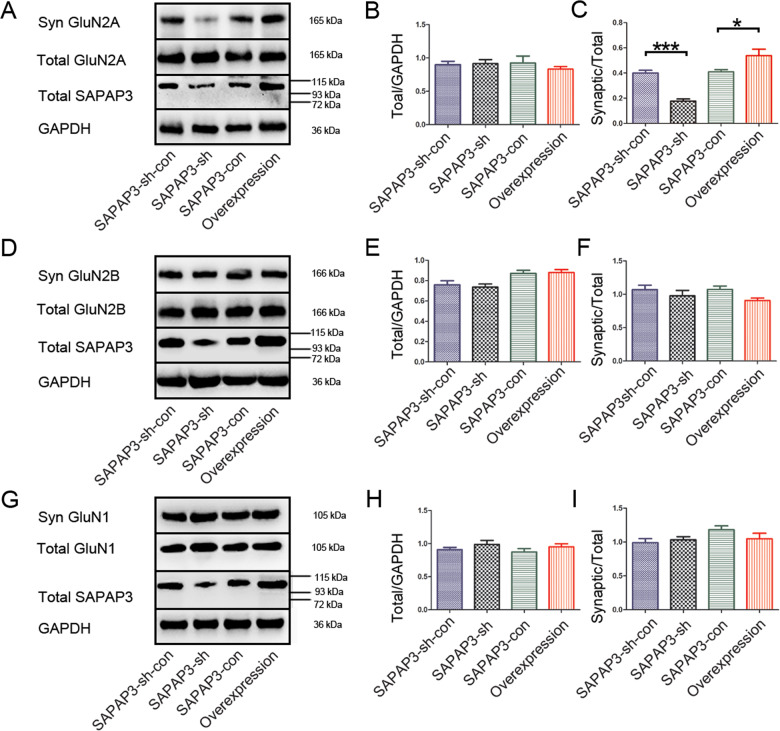
Expression of GluN1, GluN2A and GluN2B in total homogenates and the PSD after the behavioral evaluation. **A**–**C** Representative western blot of the GluN2A levels in total tissue homogenates and subcellular fractions (**A**). No significant differences in the total GluN2A levels were found among the groups (**B**). Compared with the control group, the SAPAP3-sh vector significantly decreased the synaptic/total GluN2A ratio. SAPAP3 overexpression significantly increased the synaptic/total GluN2A ratio (**C**). Representative western blots of the GluN2B (**D**) and GluN1 levels (**G**) in total tissue homogenates and subcellular fractions. No significant differences in the total GluN2B and GluN1 levels were found among the groups (**E** and **H**). The synaptic/total GluN2B and GluN1 ratios were not significantly different compared with those of the control group (**F** and **I**). GAPDH served as a loading control for the total homogenates. Data are presented as means ± SEM, *n* = 4 per group. *p < 0.05; ***p < 0.001.

PSDs, which contain receptors, signaling proteins and cytoskeletal components, are the focus of investigations of synaptic connections. Based on our findings from animal behavioral tests and electrophysiological data, the postsynaptic complexes at synapses, particularly the NMDAR-associated complex, may be perturbed. We examined the total and synaptic NMDAR levels using total lysates and biochemically purified PSD fractions from the hippocampus of mice that had been subjected to the behavioral tests to identify potential functional partners that may contribute to the animal behaviors (mice infected with SAPAP3-sh had a longer latency and a lower seizure frequency compared with the control mice) and electrophysiological findings (mice brain slices infected with SAPAP3-sh had a lower mEPSC amplitude/frequency than the control slices). GluN2A, the “adult” subunit of NMDAR, was significantly affected. No significant difference in the total levels of the GluN2A protein was observed among the groups. The ratio of synaptic to total GluN2A was significantly lower in the SAPAP3-sh group (0.18 ± 0.02) than in the SAPAP3-sh-con group (0.40 ± 0.02; *p* < 0.01; Fig. 6A–C). The recombinant SAPAP3 vector yielded the opposite results (SAPAP3-con group: 0.41 ± 0.02 vs. SAPAP3-overexpression group: 0.54 ± 0.05; *p* < 0.05; Fig. 6A–C). However, no significant differences in the levels of the GluN1, GluN2A or GluN2B proteins were observed in the total hippocampal lysates. The synaptic/total GluN2B and GluN1 ratios were not significantly different among the groups (*p* > 0.05; Fig. 6D–I). In general, GluN1/GluN2A channels have a higher open probability than GluN1/GluN2B channels [32]. The upregulation of GluN2A-containing NMDARs augmented NMDARs-mediated currents [33, 34], which may be related to our previous in vitro electrophysiological data.

### Changes in the SAPAP3 levels are accompanied by morphological changes

**Fig. 7:**
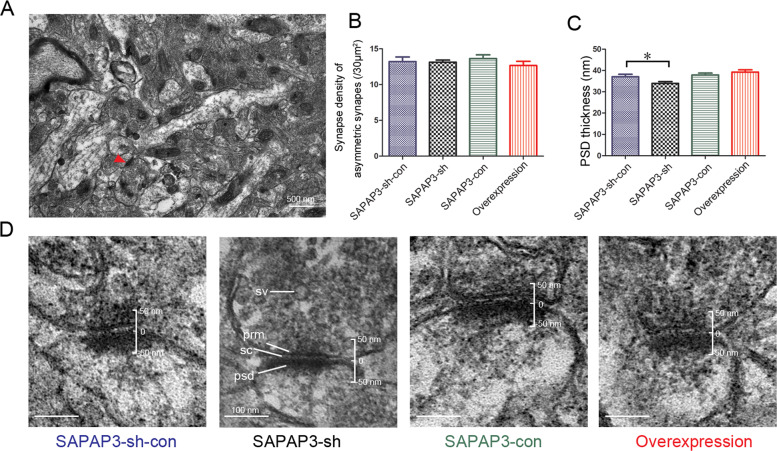
Ultrastructural morphological changes in the PSD. **A** Representative electron micrographs show the distribution of asymmetric synapses at a magnification of 25,000. The arrow shows an asymmetric synapse. **B** As shown in the bar graph, no significant difference in the density of asymmetric synapses was found among the groups (/30 μm^2^) (*n* = 24 in each group). **C** The thickness of the PSDs in the SAPAP3-sh group (*n* = 132) was significantly decreased compared with that of the PSDs in the SAPAP3-sh-con group (*n* = 110). Representative images of the different groups are shown in **D**. Synaptic vesicle (sv), postsynaptic density (PSD), presynaptic membrane (prm), synaptic clearance (sc). Data are presented as means ± SEM, *p < 0.05.

We examined the ultrastructural morphology of the PSD in the hippocampus, including the density of asymmetric synapses and the thickness of the PSD, through transmission electron microscopy to determine whether these electrophysiological and biochemical changes are accompanied by morphological changes. A 720-μm^2^ area in each group was used to quantify the synapse density. The PSD thickness was measured in 110 asymmetric synapses from the SAPAP3-sh-con group, 105 from the SAPAP3-con group, 132 from the SAPAP3-sh group and 135 from the recombinant SAPAP3 overexpression group. No significant differences in the density of asymmetric synapses were observed among the different groups (*p* > 0.05; Fig. 7B). We then examined whether perturbations in the SAPAP3 levels affected the thickness of the PSD and found that the mean thickness of the PSD in the SAPAP3-sh group was significantly less (33.98 ± 0.83 nm) than that in the SAPAP3-sh-con group (37.05 ± 1.17 nm; *p* < 0.05; Fig. 7C). The mean thickness of the PSD was greater in the SAPAP3-overexpression group than in the SAPAP3-con group, but the difference was not significant (SAPAP3 overexpression: 39.22 ± 1.09 nm vs. SAPAP3-con: 37.89 ± 0.88 nm; *p* > 0.05; Fig. 7C).

## Discussion

Despite recent advances, the understanding of drug-resistant epilepsy remains far from complete. In this study, we first observed a significant increase in SAPAP3 expression in patients with TLE and in mouse epilepsy models; second, SAPAP3 modified the behavioral phenotypes of epilepsy. In addition, excitatory transmission mediated by NMDAR was regulated by SAPAP3 in the hippocampal CA1 region. In addition, GluN2A, which is the regulatory subunit of NMDAR, was regulated by SAPAP3, and this change was accompanied by ultrastructural morphological changes in the PSD.

SAPAP3 is mainly expressed in the PSD of excitatory synapses in the striatum and is involved in the activity of ionotropic and metabotropic glutamate receptors [12]. In our study, we found that SAPAP3 expression was significantly higher in the cortex of patients with TLE than in the control subjects. Because obtaining a relatively normal hippocampus is difficult, we assessed the levels of SAPAP3 in the cortex and hippocampus of mouse epilepsy models. Similarly, we found that SAPAP3 expression was upregulated in epilepsy. Although the exact mechanism resulting in the upregulation of SAPAP3 expression in epilepsy is unclear, researchers have identified the effect of different CaMKII isoforms on SAPAP expression, which involves degradation by poly-ubiquitination and transport via MyOva [35]. The hippocampus is the most widely studied brain region in both human and experimental epilepsy investigations. The tests performed in this study focused on the hippocampus, where the function of SAPAP3 is not well understood. Considering the limitations of human studies, we used a pilocarpine-induced mouse model, which is a widely used tool for investigating TLE [36]. We used two strategies, namely, SAPAP3 knockdown and overexpression, to alter SAPAP3 activity and investigate the role of SAPAP3 in epilepsy and found that SAPAP3 overexpression decreased the latency time and increased the seizure frequency. The results showed that SAPAP3 participates in the development of epilepsy and that the upregulation of SAPAP3 expression in the mouse model and patients might be a more likely cause of epilepsy and not simply a concomitant phenomenon.

It is generally accepted that epilepsy results from impairments in GABAergic and glutamatergic neurotransmission [4, 5]. In addition, TLE is characterized by hyperexcitability of hippocampal neurons [37], and SAPAP3 increases the initial formation of excitatory synapses [11]. An electrophysiological study was performed to directly assess the effects of SAPAP3 on neuronal hyperexcitability and confirm the data from the animal behavioral tests. Perturbations in SAPAP3 significantly affected excitatory synaptic transmission as reflected by the frequencies and amplitudes of mEPSCs but not inhibitory synaptic transmission, which is consistent with previous studies of the striatum [12, 35, 38]. Excitatory synaptic transmission, which is mediated by activation of AMPA-type and NMDA-type glutamate receptors, is a central mechanism for controlling neuronal activity, including epilepsy [39]. We recorded the evoked EPSCs after infection with SAPAP3-sh or the SAPAP3-overexpression virus and found that SAPAP3 exerted an obvious effect on NMDAR-mediated currents. A study conducted by Welch and colleagues revealed that SAPAP3 participates in AMPAR- and NMDAR-mediated transmission in the striatum, but the CA3-CA1 regions of the hippocampus of SAPAP3^−^/^−^ mice show no defects in basal synaptic transmission SAPAP3 [12]. This study revealed that SAPAP3 influenced the NMDA/AMPA ratio, which agrees with a previous study [12]. Interestingly, our data showed that SAPAP3 also participated in regulating AMPAR-specific currents, but the difference was not significant. Therefore, alterations in excitatory synaptic transmission associated with SAPAP3 were mainly mediated by NMDARs.

According to previous studies, scaffolding proteins in the PSD play a key organizational role in regulating the kinetics of AMPARs and NMDARs and thereby affect the efficacy of synaptic transmission, which is important for brain function, including epilepsy [40]. In addition, scaffolding proteins constantly modify transmission efficiencies by regulating local protein synthesis in dendrites in response to neuronal activity. As shown in our data, NMDAR-mediated currents play an important role after regulating the changes in SAPAP3 during epilepsy. We examined the synaptic levels of NMDAR proteins using biochemically purified PSD fractions from the hippocampi of different groups obtained after the behavioral tests to investigate a potential role for SAPAP3 in postsynaptic assembly. Functional NMDARs are assembled from two constitutive GluN1 subunits: two GluN2A-D glutamate-binding subunits, which modulate the functional properties of the ion channel [41, 42], and GluN2A and GluN2B subunits, which are the predominant GluN2 subunits in the adult temporal cortex and hippocampus [43], the regions involved in limbic epilepsy. Therefore, the levels of GluN1, GluN2A and GluN2B were examined in our study. We found that synaptic GluN2A was increased in the SAPAP3-overexpression group and decreased in the SAPAP3-sh group compared with the levels in the respective control groups. No significant differences were observed in the total GluN1, GluN2A and GluN2B levels, which is consistent with other studies [12]. However, the GluN1 subunit, as an obligatory subunit, is needed for the formation of functional NMDA receptor channels [44], and our data showed no significant difference in synaptic GluN1, partly because some GluN1 subunits were not assembled with GluN2 subunits [45] or due to other possibilities. Membrane proteins are trafficked between the plasma membrane and intracellular compartments via vesicle-mediated exocytosis (insertion) and endocytosis (internalization). The precise molecular events involving SAPAP3 that regulate synaptic NMDARs are unknown and require intense investigation.

Previous studies have shown that excessive Ca2^+^ influx through NMDA receptors leads to many pathophysiological consequences, including epileptiform activities and neurodegeneration [46, 47]. GluN2A and GluN2B are both necessary for normal performance in a wide range of behavioral procedures spanning cognitive, emotional and motor function [48, 49]; however, the remarkable functional heterogeneity of NMDARs mainly results from differences in the composition of GluN2 subunits [41]. In epilepsy, the activation of GluN2A-containing NMDARs is necessary for epileptogenesis, and GluN2A activates a distinct intracellular signaling pathway that is linked to brain-derived neurotrophic factor (BDNF) expression and then contributes to the development of epilepsy [50]. However, some differences were observed in the biochemical and electrophysiological findings compared with those in the striatum, which may be due to the different models and the possible compensatory functions of other SAPAP family members that are highly expressed in the hippocampus [12, 20]. In general, the ultrastructural properties of the PSD and the electrophysiological findings showed that SAPAP3 participates in organizing the PSD. Based on the ultrastructural morphological differences in the PSD, GluN2A may be one of the subunits involved in this process.

In summary, the results of our electrophysiological study combined with the biochemical findings provide evidence for the participation of SAPAP3 in epilepsy, which is possibly related to NMDARs, particularly synaptic GluN2A.

## Supplementary information

Supplementary Fig. 1, Supplementary Fig. 2, Supplementary Table 1, Supplementary Table 2

AJ-Checklist

Consent responses from all authors to author list.

Full length uncropped original western blots

## Data Availability

The datasets generated and analyzed during this study are available from the corresponding author on reasonable request.
