# Supplementary material for: SAPAP3 regulates epileptic seizures involving GluN2A in post-synaptic densities
Source: Cell Death Dis. 2022 May 5;13(5):437. doi: 10.1038/s41419-022-04876-9 (PMC9072407; doi:10.1038/s41419-022-04876-9)
Supplement: Supplementary file 1 — Supplementary Fig. 1, Supplementary Fig. 2, Supplementary Table 1, Supplementary Table 2 [file 41419_2022_4876_MOESM1_ESM.docx]

**
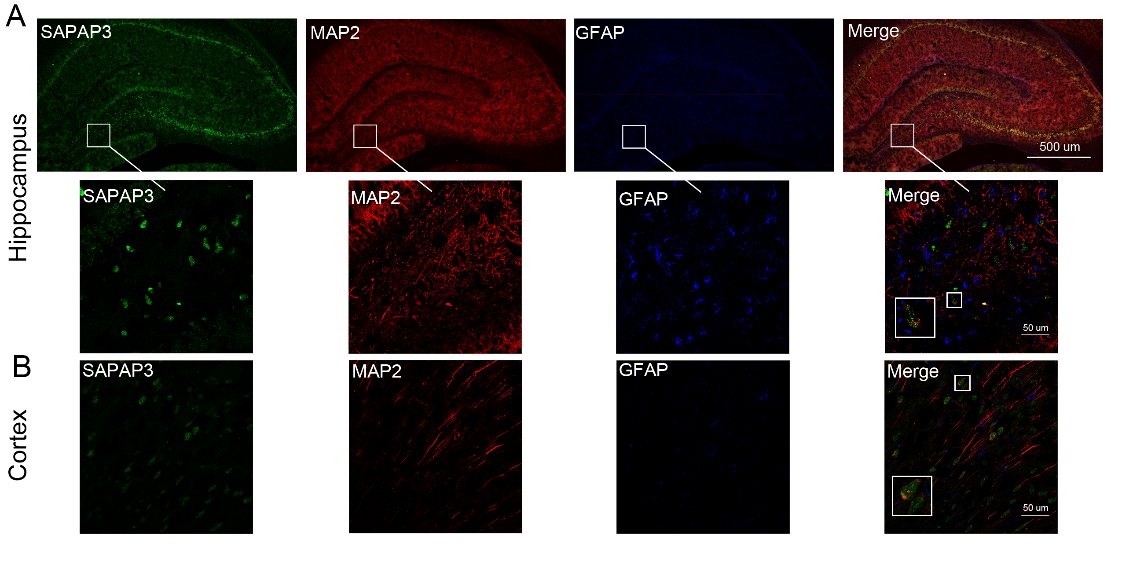
Fig. S1 Localization of SAPAP3 in the control brain. (A and B)** Triple-label immunofluorescence showed that SAPAP3 (green) and GFAP (blue) were not co-expressed in astrocytes, but SAPAP3 (green) and MAP2 (red) were mainly coexpressed within mouse hippocampal and cortical neurons.


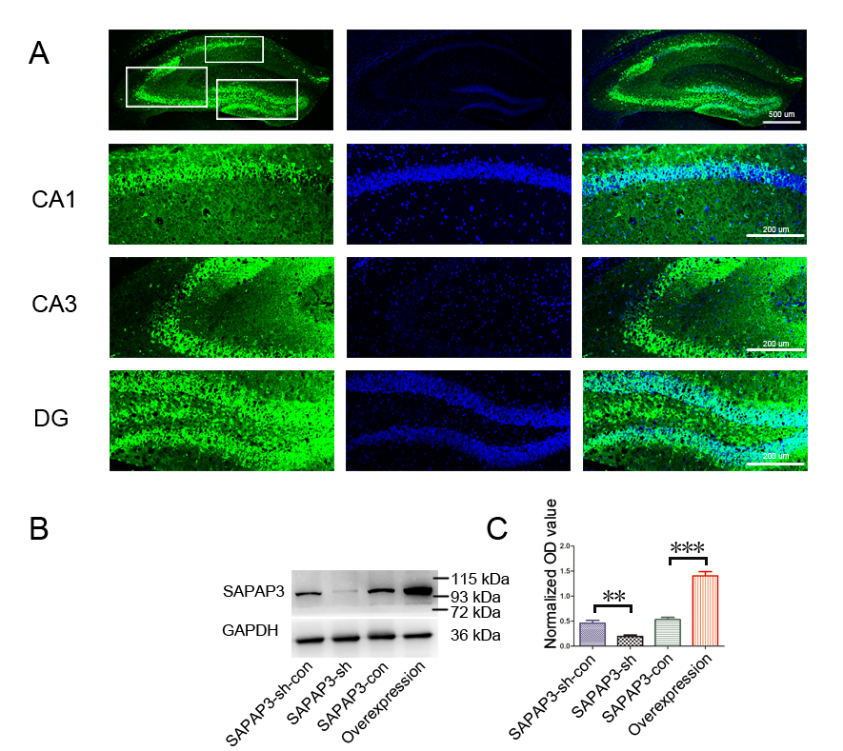


**Fig. S2 Confirmation of the infection efficiency in the hippocampus of mice that had been injected with the lentivirus vectors. (A)** Immunofluorescence images showing the distribution of GFP (green) in the hippocampus 7 days after injection. DAPI is shown in blue. **(B and C)** Representative western blots of SAPAP3 expression in the hippocampus of the different groups 7 days after injection. Comparison of the mean immunoblotting intensity among the different groups. Data are presented as means ± SEM, *n*=5 per group. ***P*<0.01; ****P*<0.001.

**Supplemental Table 1** Clinical characteristics of TLE patients and control patients.

| Patients | Age (y) | Sex (M/F) | Course (y) | AEDs before surgery | Pathology | Side of resected temporal lobe |  |
| --- | --- | --- | --- | --- | --- | --- | --- |
| E1 | 27 | M | 10 | CBZ, VPA, PB, TPM | Gliosis | R |  |
| E2 | 22 | F | 11 | OXC, CBZ, VPA, PHT | NL | R |  |
| E3 | 35 | F | 12 | LTG, TPM, PHT | NL | R |  |
| E4 | 35 | F | 14 | CBZ, LTG, TPM, PHT | Gliosis | R |  |
| E5 | 20 | M | 13 | VPA, CBZ, PB, PHT, LEV | NL | L |  |
| E6 | 30 | F | 8 | PB, CBZ, LEV, VPA, PHT | NL | L |  |
| E7 | 27 | M | 8 | CBZ, PHT, TPM | NL, Gliosis | R |  |
| E8 | 25 | M | 12 | PHT, CBZ, PB, VPA | Gliosis | R |  |
| E9 | 22 | M | 14 | CBZ, VPA, PB, TPM | Gliosis | R |  |
| E10 | 32 | M | 12 | CBZ, VPA, TPM, LEV | NL | L |  |
| E11 | 47 | M | 23 | OXC, VPA, CBZ, PHT | Gliosis | R |  |
| E12 | 40 | F | 22 | VPA, CBZ, PB, PHT | NL | L |  |
| E13 | 19 | M | 7 | CBZ, VPA, PB, TPM, LTG | Gliosis | L |  |
| E14 | 33 | F | 22 | PHT, PB, VPA, LEV | NL | L |  |
| E15 | 26 | M | 8 | VPA, CBZ, LEV, LTG | Gliosis | R |  |
| E16 | 39 | F | 9 | LEV, VPA, PHT | NL | L |  |
| E17 | 33 | F | 7 | PHT, TPM, LTG, VPA | Gliosis | R |  |
| E18 | 23 | M | 9 | OXC, PB, CBZ, LEV | NL | R |  |
| E19 | 40 | M | 12 | PB, VPA, TPM, LEV, LTG | Gliosis | L |  |
| E20 | 31 | F | 15 | VPA, PB, TPM, LEV | NL | L |  |
| C1 | 23 | F | 0 | None | RN | R |  |
| C2 | 35 | M | 0 | None | RN | L |  |
| C3 | 26 | F | 0 | None | RN | L |  |
| C4 | 24 | M | 0 | None | RN | R |  |
| C5 | 38 | F | 0 | None | RN | R |  |
| C6 | 43 | M | 0 | None | RN | R |  |
| C7 | 25 | F | 0 | None | RN | L |  |
| C8 | 18 | M | 0 | None | RN | R |  |
| C9 | 15 | F | 0 | None | RN | R |  |
| C10 | 19 | M | 0 | None | RN | R |  |

E epilepsy, C control, F female, M male, y year, AEDs antiepileptic drugs, VPA valproic acid, PB phenobarbital, CBZ carbamazepine, PHT phenytoin, GBP gabapentin, LTG lamotrigine, TPM topiramate, LEV levetiracetam, OXC oxcarbazepine, L left, R right, NL neuron loss, RN relative normal.

**Supplemental Table 2** Sense oligomers of targeting the mouse SAPAP3 gene.

| symbol | Strand sequence 5′–3′ | titer of the lentivirus (TU/mL) |
| --- | --- | --- |
| LV-SAPAP3-sh1 | AGTGGTTCATCAAGATGCT | 5 × 10^8^ |
| LV-SAPAP3-sh2 | CAGCTAAGCGAAGAGTTTA | 5 × 10^8^ |
| LV-SAPAP3-sh3 | CCAGGACTTATCACTATTT | 8 × 10^8^ |
| SAPAP3-sh-con | TTCTCCGAACGTGTCACGT | 2 × 10^9^ |
| SAPAP3-con |  | 2 × 10^9^ |
| Recombinant-SAPAP3 | NM_198618 | 5 × 10^8^ |
