# Supplementary material for: SAPAP3 regulates epileptic seizures involving GluN2A in post-synaptic densities
Source: Cell Death Dis. 2022 May 5;13(5):437. doi: 10.1038/s41419-022-04876-9 (PMC9072407; doi:10.1038/s41419-022-04876-9)
Supplement: Supplementary file 3 — Consent responses from all authors to author list. [file 41419_2022_4876_MOESM3_ESM.pdf]

Re:Consent form for Final Author List.

"zhang yanke" <yyykzhang@126.com>

收件人：xfyp1218 <xfyp1218@163.com>

时　间：2022-4-21 13:06:21

附　件：

Checked and approved. Many thanks for the hard work of all of the co-authors.

Kind regards
Yanke

zhang yanke

yyykzhang@126.com

签名由 网易邮箱大师 定制

On 04/21/2022 10:38，xfyp1218<xfyp1218@163.com> wrote：

Manuscript CDDIS-21-1312RR
Title: SAPAP3 regulates epileptic seizures involving GluN2A in post-synaptic densities.

Dear co-authors，

In the revision of our manuscript, we have adjusted the author list based on actual contributions, as previously discussed and agreed. Please reply to this email further to confirm your approval of the final author list as follows:

Final author list: Yanke Zhang[†], Junhong Wu[†], Yin Yan[†], Yixue Gu, Yuanlin Ma, Min Wang, Hui Zhang, Kaiyan Tao, Yang Lü, Weihua Yu, Wei Jing*, Xuefeng Wang*, Xin Tian*.

[†]These authors contributed equally to this work.
***Corresponding author.**

Many thanks to all of you for your contributions to this work.

Yours sincerely,
Xuefeng Wang

Re:Consent form for Final Author List.

"Junhong wu" <wu_junhong2021@163.com>

收件人：xfyp1218 <xfyp1218@163.com>

时　间：2022-4-21 11:10:38

附　件：

Checked and agreed. Many thanks.

Best regards
Junhong

Junhong wu

wu_junhong2021@163.com

签名由 网易邮箱大师 定制

On 04/21/2022 10:38，xfyp1218<xfyp1218@163.com> wrote：

Manuscript CDDIS-21-1312RR
Title: SAPAP3 regulates epileptic seizures involving GluN2A in post-synaptic densities.

Dear co-authors，

In the revision of our manuscript, we have adjusted the author list based on actual contributions, as previously discussed and agreed. Please reply to this email further to confirm your approval of the final author list as follows:

Final author list: Yanke Zhang[†], Junhong Wu[†], Yin Yan[†], Yixue Gu, Yuanlin Ma, Min Wang, Hui Zhang, Kaiyan Tao, Yang Lü, Weihua Yu, Wei Jing*, Xuefeng Wang*, Xin Tian*.

[†]These authors contributed equally to this work.
***Corresponding author.**

Many thanks to all of you for your contributions to this work.

Yours sincerely,
Xuefeng Wang

Re:Consent form for Final Author List.

"yan yin" <yanyin_585@163.com>

收件人：xfyp1218 <xfyp1218@163.com>

时　间：2022-4-21 13:21:44

附　件：

Checked and agreed. Thanks to all of the the co-authors for the contribution they have made on this paper.

Regards
Yin Yan

yan yin

yanyin_585@163.com

签名由 网易邮箱大师 定制

On 04/21/2022 10:38，xfyp1218<xfyp1218@163.com> wrote：

Manuscript CDDIS-21-1312RR
Title: SAPAP3 regulates epileptic seizures involving GluN2A in post-synaptic densities.

Dear co-authors，

In the revision of our manuscript, we have adjusted the author list based on actual contributions, as previously discussed and agreed. Please reply to this email further to confirm your approval of the final author list as follows:

Final author list: Yanke Zhang†, Junhong Wu†, Yin Yan†, Yixue Gu, Yuanlin Ma, Min Wang, Hui Zhang, Kaiyan Tao, Yang Lü, Weihua Yu, Wei Jing*, Xuefeng Wang*, Xin Tian*.

†These authors contributed equally to this work.
***Corresponding author.**

Many thanks to all of you for your contributions to this work.

Yours sincerely,
Xuefeng Wang

Re:Consent form for Final Author List.

"谷依雪" <guyixue108@163.com>
收件人：xfyp1218 <xfyp1218@163.com>
时 间：2022-4-21 12:53:06
附 件：

Thanks for your email.

Checked and approved.

Sincerely,
Yixue Gu

At 2022-04-21 10:38:54, "xfyp1218" <xfyp1218@163.com> wrote:

> Manuscript CDDIS-21-1312RR
> Title: SAPAP3 regulates epileptic seizures involving GluN2A in post-synaptic densities.
>
> Dear co-authors,
>
> In the revision of our manuscript, we have adjusted the author list based on actual contributions, as previously discussed and agreed. Please reply to this email further to confirm your approval of the final author list as follows:
>
> **Final author list: Yanke Zhang†, Junhong Wu†, Yin Yan†, Yixue Gu, Yuanlin Ma, Min Wang, Hui Zhang, Kaiyan Tao, Yang Lü, Weihua Yu, Wei Jing*, Xuefeng Wang*, Xin Tian*.**
>
> **†These authors contributed equally to this work.**
> ***Corresponding author.**
>
> Many thanks to all of you for your contributions to this work.
>
> Yours sincerely,
> Xuefeng Wang

Re:Consent form for Final Author List.

"Ma Yuanlin" <ylinma@126.com>

收件人：xfyp1218 <xfyp1218@163.com>

时　间：2022-4-21 11:42:03

附　件：

Dear Prof Wang,

I have checked and approved the author list. Many thank to all of the co-authors for their collaboration and contribution.

Regards
Yuanlin

Ma Yuanlin

ylinma@126.com

签名由 网易邮箱大师 定制

On 04/21/2022 10:38，xfyp1218<xfyp1218@163.com> wrote：

Manuscript CDDIS-21-1312RR
Title: SAPAP3 regulates epileptic seizures involving GluN2A in post-synaptic densities.

Dear co-authors，

In the revision of our manuscript, we have adjusted the author list based on actual contributions, as previously discussed and agreed. Please reply to this email further to confirm your approval of the final author list as follows:

Final author list: Yanke Zhang†, Junhong Wu†, Yin Yan†, Yixue Gu, Yuanlin Ma, Min Wang, Hui Zhang, Kaiyan Tao, Yang Lü, Weihua Yu, Wei Jing*, Xuefeng Wang*, Xin Tian*.

†These authors contributed equally to this work.
***Corresponding author.**

Many thanks to all of you for your contributions to this work.

Yours sincerely,
Xuefeng Wang

Re:Consent form for Final Author List.

wangmin <min12wang12@163.com>

收件人: xfyp1218 <xfyp1218@163.com>

时　间: 2022-4-21 13:12:50

附　件:

Dear Prof Wang,

I have checked the adjusted author list that we have discussed before. I can not agree more. Many thanks for your kind email.

Regards
Min Wang

wangmin

min12wang12@163.com

签名由 网易邮箱大师 定制

On 04/21/2022 10:38, xfyp1218<xfyp1218@163.com> wrote:

Manuscript CDDIS-21-1312RR
Title: SAPAP3 regulates epileptic seizures involving GluN2A in post-synaptic densities.

Dear co-authors,

In the revision of our manuscript, we have adjusted the author list based on actual contributions, as previously discussed and agreed. Please reply to this email further to confirm your approval of the final author list as follows:

Final author list: Yanke Zhang[†], Junhong Wu[†], Yin Yan[†], Yixue Gu, Yuanlin Ma, Min Wang, Hui Zhang, Kaiyan Tao, Yang Lü, Weihua Yu, Wei Jing*, Xuefeng Wang*, Xin Tian*.

[†]These authors contributed equally to this work.
***Corresponding author.**

Many thanks to all of you for your contributions to this work.

Yours sincerely,
Xuefeng Wang

Re:Consent form for Final Author List.

"Hui Zhang" <hui_zhang0208@163.com>

收件人：xfyp1218 <xfyp1218@163.com>

时　间：2022-4-21 15:35:06

附　件：

Dear Xuefeng,

Thanks for your mail. I have checked and confirmed this author list.

Should you have further concerns, please do not hesitate to contact me.

All my best wishes,
Hui Zhang, M.D.

At 2022-04-21 10:38:54, "xfyp1218" <xfyp1218@163.com> wrote:

> Manuscript CDDIS-21-1312RR
> Title: SAPAP3 regulates epileptic seizures involving GluN2A in post-synaptic densities.
>
> Dear co-authors,
>
> In the revision of our manuscript, we have adjusted the author list based on actual contributions, as previously discussed and agreed. Please reply to this email further to confirm your approval of the final author list as follows:
>
> **Final author list: Yanke Zhang†, Junhong Wu†, Yin Yan†, Yixue Gu, Yuanlin Ma, Min Wang, Hui Zhang, Kaiyan Tao, Yang Lü, Weihua Yu, Wei Jing*, Xuefeng Wang*, Xin Tian*.**
>
> **†These authors contributed equally to this work.**
> ***Corresponding author.**
>
> Many thanks to all of you for your contributions to this work.
>
> Yours sincerely,
> Xuefeng Wang

Re:Consent form for Final Author List.

kaiyan <taokai_yan@126.com>

收件人: xfyp1218 <xfyp1218@163.com>

时　间: 2022-4-21 15:55:08

附　件:

Thank you for your letter and reminder.

Thanks to our team for the efforts, and I agree with the author list.

Sincerely,
Kaiyan

At 2022-04-21 10:38:54, "xfyp1218" <xfyp1218@163.com> wrote:

> Manuscript CDDIS-21-1312RR
> Title: SAPAP3 regulates epileptic seizures involving GluN2A in post-synaptic densities.
>
> Dear co-authors,
>
> In the revision of our manuscript, we have adjusted the author list based on actual contributions, as previously discussed and agreed. Please reply to this email further to confirm your approval of the final author list as follows:
>
> **Final author list: Yanke Zhang†, Junhong Wu†, Yin Yan†, Yixue Gu, Yuanlin Ma, Min Wang, Hui Zhang, Kaiyan Tao, Yang Lü, Weihua Yu, Wei Jing*, Xuefeng Wang*, Xin Tian*.**
>
> **†These authors contributed equally to this work.**
> ***Corresponding author.**
>
> Many thanks to all of you for your contributions to this work.
>
> Yours sincerely,
> Xuefeng Wang

Re:Consent form for Final Author List.

"吕洋" <yanglyu@hospital.cqmu.edu.cn>
收件人: xfyp1218 <xfyp1218@163.com>
时　间: 2022-4-21 15:45:33
附　件:

Dear Prof. Wang,

I agree with the Final Author List of the Manuscript CDDIS-21-1312RR, thanks a lot!

Best Regards!

Yang Lü, *M.D., Ph.D*
Professor and Vice Director
Department of Geriatrics, The First Affiliated Hospital of Chongqing Medical University
1 Youyi Road, Yuzhong District, Chongqing 400016, China
Office: (86)-23-89011632
Fax: (86)-23-68811487

吕洋
教授/主任医师 博士生导师 科副主任
重庆医科大学附属第一医院老年病科
地址：重庆市渝中区友谊路1号（400016）
电话：023-89011632
传真：023-68811487

------------------ Original ------------------

From: "xfyp1218"<xfyp1218@163.com>;
Date: Thu, Apr 21, 2022 10:39 AM

To: "yyykzhang"<yyykzhang@126.com>; "wu_junhong2021"<wu_junhong2021@163.com>; "yanyin_585" <yanyin_585@163.com>; "guyixue108"<guyixue108@163.com>; "ylinma"<ylinma@126.com>; "min12wang12"<min12wang12@163.com>; "hui_zhang0208"<hui_zhang0208@163.com>; "taokai_yan" <taokai_yan@126.com>; "yanglyu"<yanglyu@hospital.cqmu.edu.cn>; "余维华"<yuweihua@cqmu.edu.cn>; "jingweistar"<jingweistar@163.com>; "xintian"<xintian@cqmu.edu.cn>;
Subject: Consent form for Final Author List.

Manuscript CDDIS-21-1312RR
Title: SAPAP3 regulates epileptic seizures involving GluN2A in post-synaptic densities.

Dear co-authors,

In the revision of our manuscript, we have adjusted the author list based on actual contributions, as previously discussed and agreed. Please reply to this email further to confirm your approval of the final author list as follows:

Final author list: Yanke Zhang†, Junhong Wu†, Yin Yan†, Yixue Gu, Yuanlin Ma, Min Wang, Hui Zhang, Kaiyan Tao, Yang Lü, Weihua Yu, Wei Jing*, Xuefeng Wang*, Xin Tian*.

†These authors contributed equally to this work.
***Corresponding author.**

Many thanks to all of you for your contributions to this work.

Yours sincerely,
Xuefeng Wang

Re:Consent form for Final Author List.

"余维华" <yuweihua@cqmu.edu.cn>

收件人: xfyp1218 <xfyp1218@163.com>

时 间: 2022-4-21 15:42:46

附 件:

Dear Prof. Wang,

I agree with the Final Author List, thanks a lot!

Best Regards!

Weihua Yu

------------------ Original ------------------

From: "xfyp1218"<xfyp1218@163.com>;
Date: Thu, Apr 21, 2022 10:39 AM
To: "yyykzhang"<yyykzhang@126.com>; "wu_junhong2021"<wu_junhong2021@163.com>; "yanyin_585"<yanyin_585@163.com>; "guyixue108"<guyixue108@163.com>; "ylinma"<ylinma@126.com>; "min12wang12"<min12wang12@163.com>; "hui_zhang0208"<hui_zhang0208@163.com>; "taokai_yan"<taokai_yan@126.com>; "吕洋"<yanglyu@hospital.cqmu.edu.cn>; "yuweihua"<yuweihua@cqmu.edu.cn>; "jingweistar"<jingweistar@163.com>; "xintian"<xintian@cqmu.edu.cn>;
Subject: Consent form for Final Author List.

Manuscript CDDIS-21-1312RR

Title: SAPAP3 regulates epileptic seizures involving GluN2A in post-synaptic densities.

Dear co-authors,

In the revision of our manuscript, we have adjusted the author list based on actual contributions, as previously discussed and agreed. Please reply to this email further to confirm your approval of the final author list as follows:

Final author list: Yanke Zhang†, Junhong Wu†, Yin Yan†, Yixue Gu, Yuanlin Ma, Min Wang, Hui Zhang, Kaiyan Tao, Yang Lü, Weihua Yu, Wei Jing*, Xuefeng Wang*, Xin Tian*.

†These authors contributed equally to this work.
***Corresponding author.**

Many thanks to all of you for your contributions to this work.

Yours sincerely,

Xuefeng Wang

Re: Consent form for Final Author List.

"景玮" <jingweistar@163.com>

收件人：xfyp1218 <xfyp1218@163.com>

时　间：2022-4-21 14:14:05

附　件：

Dear Professor Wang,
Thanks for your email. I have checked the final author list and agreed with it. Thanks to all authors for their contributions.
Yours turly,
Wei Jing

---- 回复的原邮件 ----
发件人 xfyp1218<xfyp1218@163.com> 日期 2022年04月21日 10:38 收件人 yyykzhang@126.com<yyykzhang@126.com>、wu_junhong2021@163.com<wu_junhong2021@163.com>、yanyin_585@163.com<yanyin_585@163.com>、guyixue108@163.com<guyixue108@163.com>、ylinma@126.com<ylinma@126.com>、min12wang12@163.com<min12wang12@163.com>、hui_zhang0208@163.com<hui_zhang0208@163.com>、taokai_yan@126.com<taokai_yan@126.com>、yanglyu@hospital.cqmu.edu.cn<yanglyu@hospital.cqmu.edu.cn>、yuweihua@cqmu.edu.cn<yuweihua@cqmu.edu.cn>、jingweistar@163.com<jingweistar@163.com>、xintian@cqmu.edu.cn<xintian@cqmu.edu.cn> 抄送至 主题 Consent form for Final Author List.

Manuscript CDDIS-21-1312RR
Title: SAPAP3 regulates epileptic seizures involving GluN2A in post-synaptic densities.

Dear co-authors,

In the revision of our manuscript, we have adjusted the author list based on actual contributions, as previously discussed and agreed. Please reply to this email further to confirm your approval of the final author list as follows:

Final author list: Yanke Zhang†, Junhong Wu†, Yin Yan†, Yixue Gu, Yuanlin Ma, Min Wang, Hui Zhang, Kaiyan Tao, Yang Lü, Weihua Yu, Wei Jing*, Xuefeng Wang*, Xin Tian*.
†These authors contributed equally to this work.
***Corresponding author.**

Many thanks to all of you for your contributions to this work.

Yours sincerely,
Xuefeng Wang

Re:Consent form for Final Author List.

"Xin Tian" <xintian@cqmu.edu.cn>

收件人：xfyp1218 <xfyp1218@163.com>

时　间：2022-4-21 10:47:24

附　件：

Checked and agreed.

Kind regards,
Xin Tian

------------------ Original ------------------

From: "xfyp1218"<xfyp1218@163.com>;
Date: Thu, Apr 21, 2022 10:38 AM
To: "yyykzhang"<yyykzhang@126.com>; "wu_junhong2021"<wu_junhong2021@163.com>; "yanyin_585"<yanyin_585@163.com>; "guyixue108"<guyixue108@163.com>; "ylinma"<ylinma@126.com>; "min12wang12"<min12wang12@163.com>; "hui_zhang0208"<hui_zhang0208@163.com>; "taokai_yan"<taokai_yan@126.com>; "yanglyu"<yanglyu@hospital.cqmu.edu.cn>; "yuweihua"<yuweihua@cqmu.edu.cn>; "jingweistar"<jingweistar@163.com>; "xintian"<xintian@cqmu.edu.cn>;
Subject: Consent form for Final Author List.

Manuscript CDDIS-21-1312RR
Title: SAPAP3 regulates epileptic seizures involving GluN2A in post-synaptic densities.

Dear co-authors,

In the revision of our manuscript, we have adjusted the author list based on actual contributions, as previously discussed and agreed. Please reply to this email further to confirm your approval of the final author list as follows:

Final author list: Yanke Zhang†, Junhong Wu†, Yin Yan†, Yixue Gu, Yuanlin Ma, Min Wang, Hui Zhang, Kaiyan Tao, Yang Lü, Weihua Yu, Wei Jing*, Xuefeng Wang*, Xin Tian*.

†These authors contributed equally to this work.
***Corresponding author.**

Many thanks to all of you for your contributions to this work.

Yours sincerely,
Xuefeng Wang
