# Supplementary material for: SAPAP3 regulates epileptic seizures involving GluN2A in post-synaptic densities
Source: Cell Death Dis. 2022 May 5;13(5):437. doi: 10.1038/s41419-022-04876-9 (PMC9072407; doi:10.1038/s41419-022-04876-9)

Fig. 1B full blots

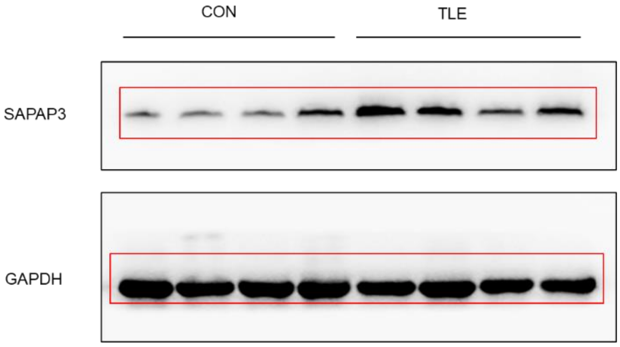

Fig. 2B full blots

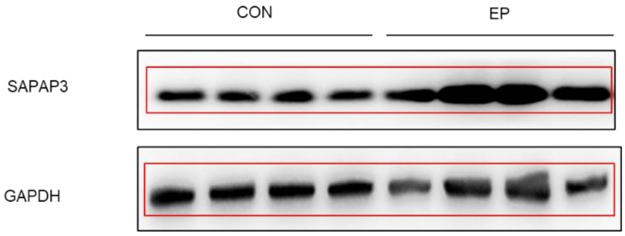

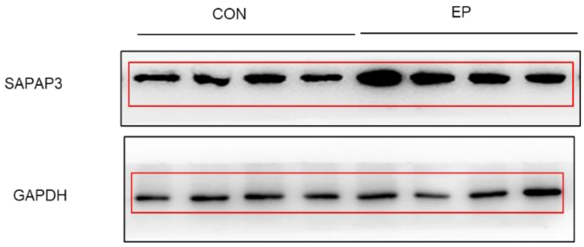

Fig. 2C full blots

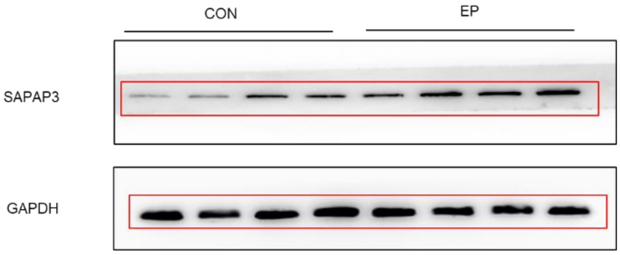

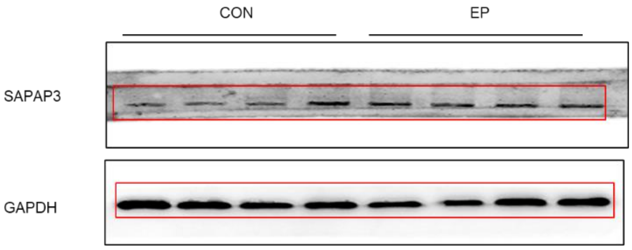

Fig. 6A full blots

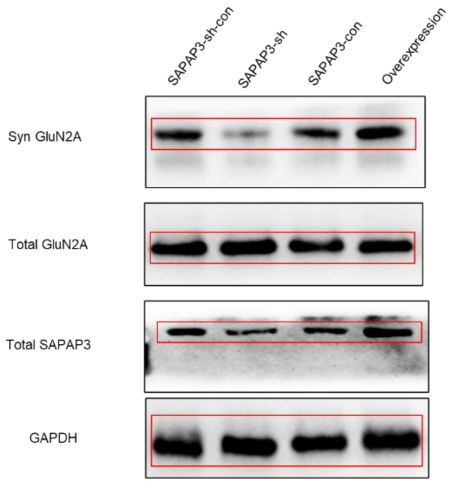

Fig. 6B full blots

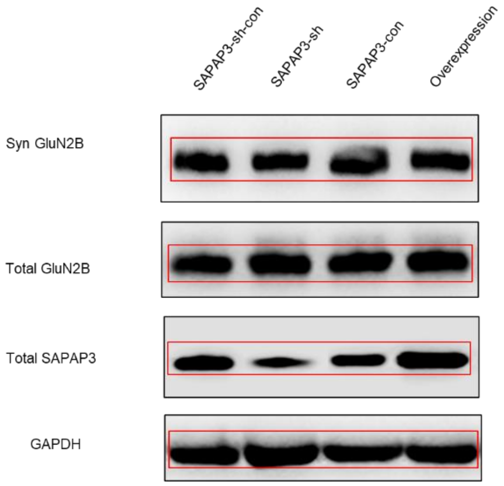

Fig. 6C full blots

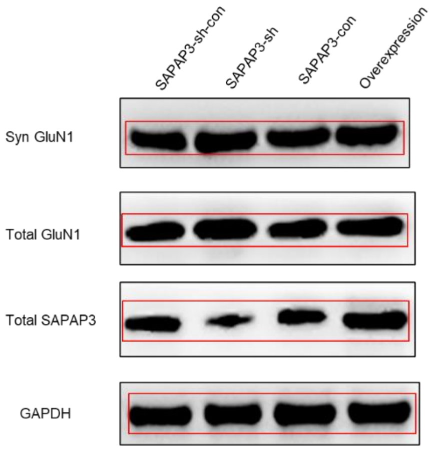

Fig. S2 full blots

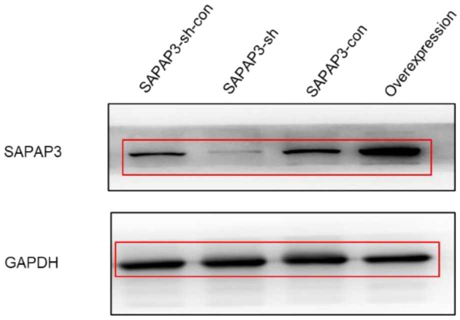

Supplement: Supplementary file 4 — Full length uncropped original western blots [file 41419_2022_4876_MOESM4_ESM.pdf]
